# Concomitant Pulmonary Aspergillosis Following Severe COVID-19

**DOI:** 10.7759/cureus.41112

**Published:** 2023-06-28

**Authors:** Ahmad Munir, Hadi Dahhan, Zoha Huda, Nazir A Lone

**Affiliations:** 1 Radiology, Peconic Bay Medical Center, Riverhead, USA; 2 Radiology, Plainview Hospital, Plainview, USA; 3 Critical Care, Peconic Bay Medical Center, Riverhead, USA

**Keywords:** invasive pulmonary aspergillosis, opportunistic infection, corticosteroid therapy, cavitary lung disease, pneumonia, covid-19

## Abstract

This case report discusses an atypical complication of COVID-19 pneumonia in a 68-year-old male patient, distinguished by the development of cavitary lung disease and a subsequent incidence of invasive pulmonary aspergillosis (IPA). This adverse development transpired following a prolonged hospitalization and an extensive course of corticosteroid therapy post-COVID-19 pneumonia. This case accentuates the importance of vigilance in observing patients with severe COVID-19 pneumonia for potential opportunistic infections, particularly given the inherent risks associated with prolonged corticosteroid therapy. Prompt diagnosis and initiation of treatment are key to enhancing patient outcomes in such presentations.

## Introduction

The extensive impact of the SARS-CoV-2 virus, primarily causing COVID-19 pneumonia, has introduced an array of long-standing pulmonary symptoms and complications, prominently including chronic respiratory failure and reduced lung function [1-4]. Within this vast spectrum of complications, a rare but significant condition has emerged: the evolution of cavitary lung disease in the aftermath of severe COVID-19 pneumonia [5,6]. Further compounding the severity of this condition, these cases are prone to the development of secondary fungal or bacterial superinfections.

The use of corticosteroids, while credited for their efficacy in mitigating mortality rates and the duration of mechanical ventilation associated with COVID-19 pneumonia [7-9], has been implicated in escalating the risk of opportunistic infections, most notably in patients who are immunocompromised. Among these opportunistic infections, the manifestation of COVID-19-associated pulmonary aspergillosis has been found in up to 15% of ICU patients, according to a multinational study [10]. This condition, known as invasive pulmonary aspergillosis (IPA), is a potentially fatal fungal infection attributed to the Aspergillus species, which has been closely linked with corticosteroid use in numerous studies [11].

Given the widespread application of corticosteroids in various therapeutic scenarios, there is a need for increased awareness towards opportunistic infections such as IPA. In light of this, we present a case of IPA that exemplifies the potential complications associated with corticosteroid use.

## Case presentation

**Figure 1:**
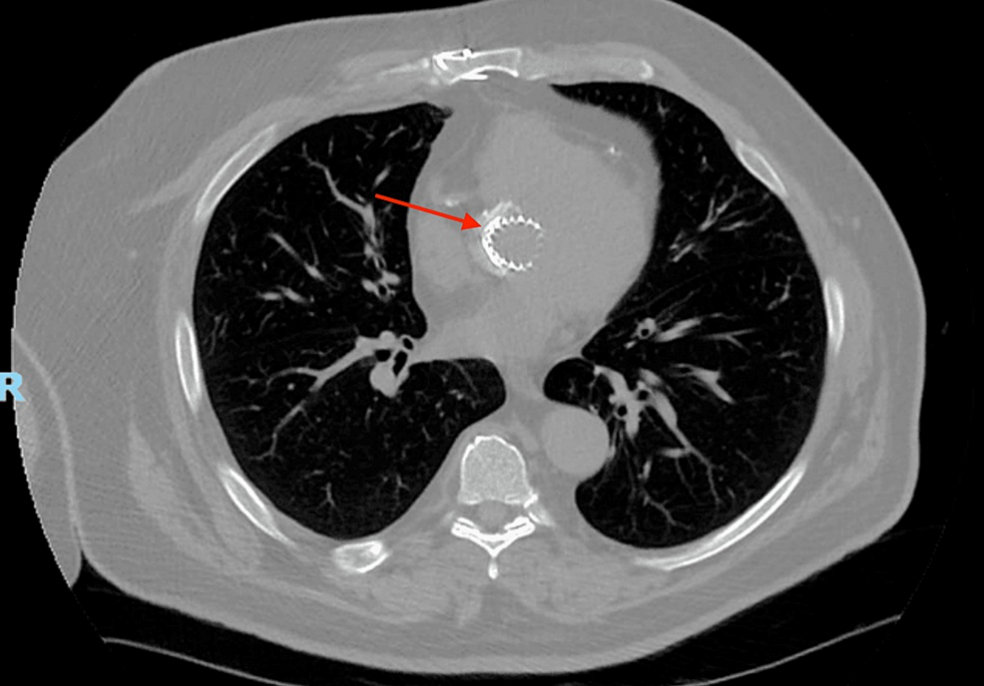
Baseline chest CT one year before COVID-19 diagnosis A computed tomography (CT) scan of the chest, conducted one year before the diagnosis of COVID-19 pneumonia, is presented herein. The axial CT image exhibits unobstructed central airways devoid of consolidations or airspace opacities. It should be noted that the patient's medical history includes a transcatheter aortic valve replacement (TAVR).

A 68-year-old male patient with a past medical history of non-insulin-dependent diabetes mellitus, grade 1 diastolic heart failure, hypertension, aortic stenosis status-post transcatheter aortic valve replacement (TAVR), hyperlipidemia, and benign prostatic hyperplasia initially presented to the emergency department due to an eight-day history of progressively worsening flu-like symptoms, shortness of breath, and fatigue. The patient was a nonsmoker with no documented prior lung disease (Figure 1). His BMI was 21.7 kg/m^2^, with a weight of 72.57 kg and a height of 182.88 cm. On initial evaluation, he was found to be febrile with a temperature of 101.5 F, tachycardic with a heart rate of 119, tachypneic with a respiratory rate of 28, and hypoxic with a pulse oximetry reading of 81% on room air. His blood pressure was 121/86. Laboratory evaluation revealed elevated inflammatory markers (D-dimer, fibrinogen, lactate dehydrogenase (LDH), C-reactive protein (CRP), and ferritin), although there was no evidence of leukocytosis or lymphocytosis. A portable chest x-ray revealed bilateral opacities, and SARS-CoV-2 by polymerase chain reaction (PCR) was positive.

The patient was hospitalized due to hypoxemic respiratory failure secondary to COVID-19 viral pneumonia. The initial treatment protocol encompassed supplemental oxygen, intravenous remdesivir (an initial loading dose of 200 mg, followed by 100 mg daily for four days), and intravenous dexamethasone (administered at 6 mg daily for 10 days). Despite these therapeutic interventions, the patient exhibited a consistent decline in oxygen saturation levels. In response, advanced respiratory support was instituted, including a high-flow nasal cannula (HFNC) and average volume-assured pressure support (AVAPS). The dosage of dexamethasone was adjusted upwards to 10 mg, administered twice daily. The patient's condition was refractory to escalated interventions and was further complicated by suspected concurrent bacterial pneumonia and notable anxiety, although microbiological investigations, including sputum and blood cultures, yielded negative results. Due to the increasing severity and refractory nature of the patient's condition, a decision was made to escalate the level of care, and the patient was subsequently transferred to the ICU 10 days post his initial admission. Given the increase in respiratory effort and persisting hypoxemia despite maximum settings on the HFNC, the patient was urgently transitioned to bilevel positive airway pressure (BiPAP) ventilation and was started on methylprednisolone administered at 40 mg intravenously every eight hours. This intervention yielded an improvement in the patient's oxygen saturation levels. To encourage compliance with non-invasive positive pressure ventilation (NIPPV), exacerbated by bouts of severe anxiety and challenges in maintaining mask fit, an infusion of dexmedetomidine was initiated. The patient remained afebrile and showed no signs of additional infection; thus, antibiotics were not administered. After six days, the patient was successfully transitioned back to the HFNC, thus obviating the need for further dexmedetomidine. In the subsequent days, the patient exhibited stable oxygen requirements and showed good tolerance to the HFNC. After a total of nine days, the patient's condition had stabilized sufficiently to permit a downgrade from the ICU to the medical ward. 

**Figure 2:**
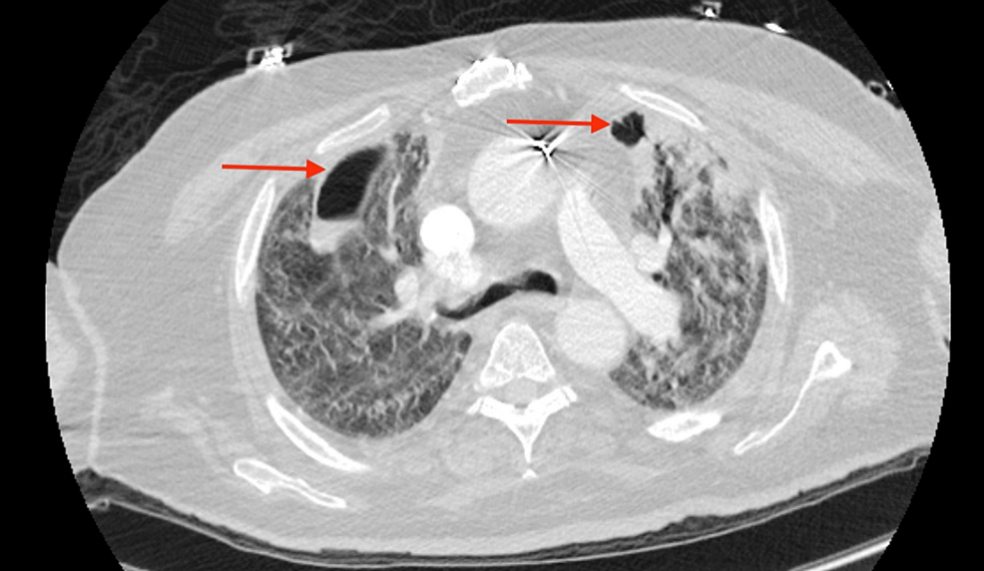
COVID-19 pneumonia complicated by superimposed cavitary bacterial pneumonia CT chest of a patient with COVID-19 pneumonia complicated by superimposed cavitary bacterial pneumonia. Axial image showing bilateral ground-glass opacities (GGOs) and multiple thick-walled cavitations involving the right middle and lower lobes.

In the medical ward, the therapeutic regimen for the patient was modified to a tapering dose of oral prednisone, starting at 60 mg per day and decreasing by 10 mg every 48 hours. Concurrently, supplemental oxygen therapy was maintained. The patient's oxygen saturation remained stable until the eighth day post-transfer from the ICU. At this juncture, the patient manifested signs of tachypnea and hypoxia, coupled with the onset of a productive cough. After these symptoms, a computed tomography (scan of the chest was conducted, uncovering bilateral cavitary lung disease (Figure 2). Notably, there was suspicion of a superimposed bacterial infection. Comprehensive microbiological investigations were performed, which included Fungitell, Galactomannan, methicillin-resistant *Staphylococcus aureus* (MRSA) nasal swab, tuberculosis (TB) gold interferon assay, sputum culture, blood culture, and urine culture. All returned negative results. Furthermore, an echocardiogram found no evidence of vegetation. The Procalcitonin level was reported to be 0.36. In response to the patient's deteriorating condition, broad-spectrum antibiotic therapy was initiated, comprising of cefepime 1 g intravenously every 12 hours for a period of five days, followed by levofloxacin 500 mg orally every 24 hours for another five days, and metronidazole 500 mg orally every eight hours for ten days. Additionally, the patient was continued on oral prednisone at 20 mg daily. Over the next 10 days, the patient's clinical status improved, with interval chest x-rays indicating a reduction in the right lung cavitary lesion and an overall improvement in infiltrates. Two weeks subsequent to the initial CT scan, a follow-up CT demonstrated a reduction in the size of the bilateral cavitary lung disease, characterized by thinner walls and an absence of debris. The patient was gradually weaned to room air and initiated on bronchodilators, namely, fluticasone-umeclidinium-vilanterol at a dose of 100 mcg-62.5 mcg-25 mcg, respectively, administered via two daily inhalations. At the time of discharge, the patient demonstrated improved respiratory function, with breathlessness only occurring during moderate activity and a cough that sporadically produced tan sputum. Following a two-month hospitalization period, the patient was discharged to a subacute rehabilitation facility.

**Figure 3:**
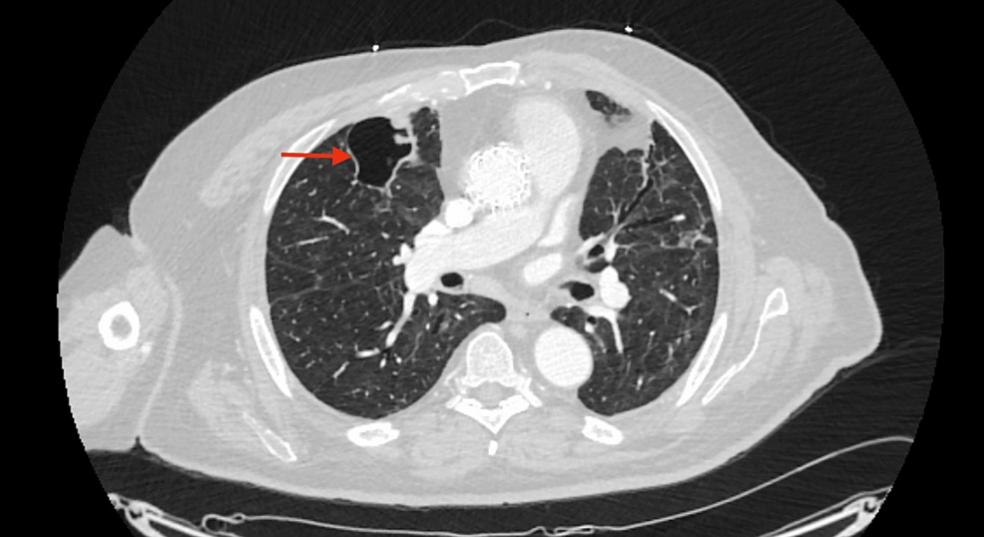
CT imaging demonstrating microbiologic resolution in a pulmonary cavity post-discharge Axial CT image of the thorax, acquired one month post discharge, exhibits a single pulmonary cavity with perceptible wall thinning and absence of intracavitary debris. The observed radiologic transformation aligns with anticipated indicators of progressive microbiologic resolution.

One week following discharge, the patient began to manifest bronchitis-like symptoms, which included a persistent cough, dyspnea, wheezing, and fatigue. The patient was managed in an outpatient setting, and the prescribed therapeutic regimen consisted of a four-week course of levofloxacin, administered orally at a daily dose of 500 mg, in conjunction with oral metronidazole, also prescribed at a daily dose of 500 mg. Moreover, the patient was placed on a tapering dosage of oral prednisone, starting at 40 mg per day and reducing by 10 mg every five days. Following one month of physical therapy and pulmonary rehabilitation, the patient demonstrated noticeable improvement. In an interval follow-up, CT imaging at one month post discharge again revealed multiple lung cavities, albeit characterized by thinner walls and devoid of debris, suggesting microbiologic resolution (Figure 3). 

**Figure 4:**
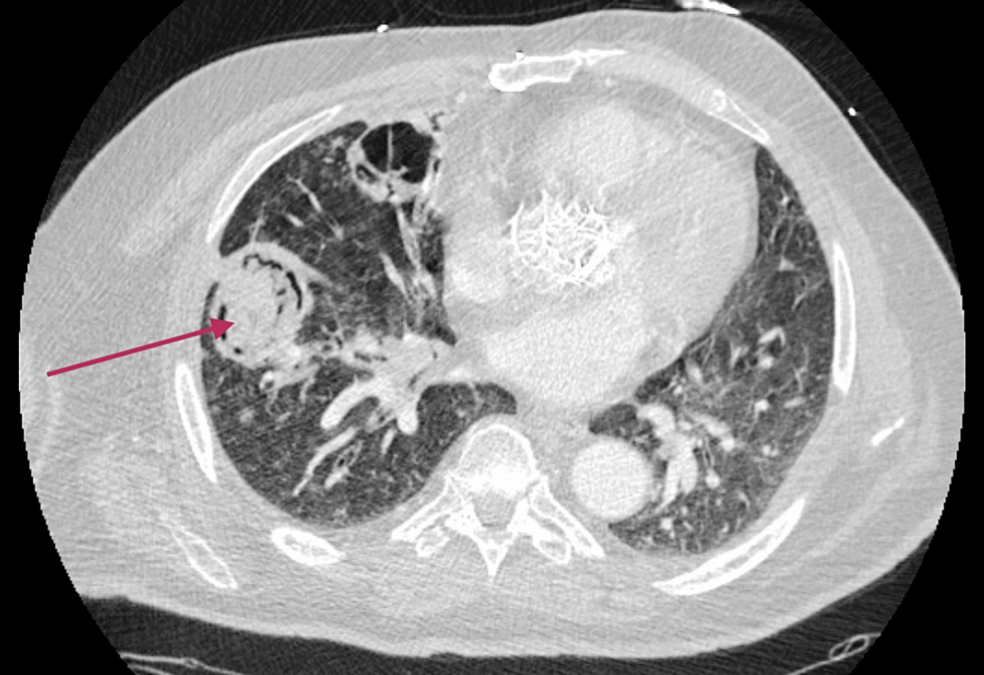
CT chest of a patient with the development of invasive pulmonary aspergillosis (IPA) and associated aspergilloma CT chest of a patient with the development of IPA as a sequela of post-COVID-19 pneumonia. Axial image showing a 4.6 x 4.2 cm cavitary lesion in the right lower lobe with intracavitary mass/debris representing an aspergilloma.

**Figure 5:**
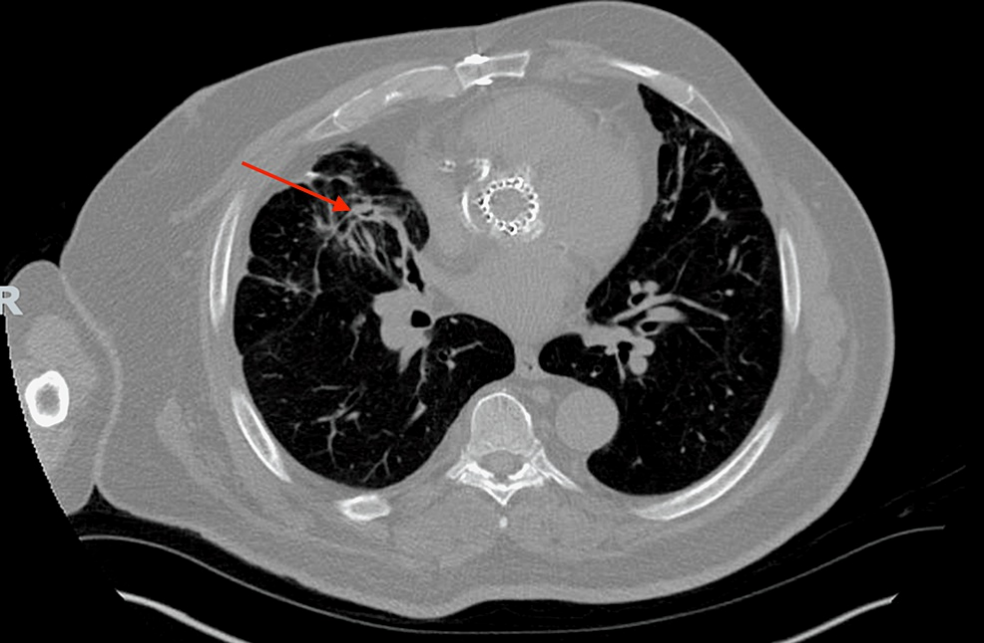
Post-operative CT imaging following right lower lung wedge resection Axial CT image showcasing the thoracic region after the patient underwent a right lower lobe wedge resection.

**Figure 6:**
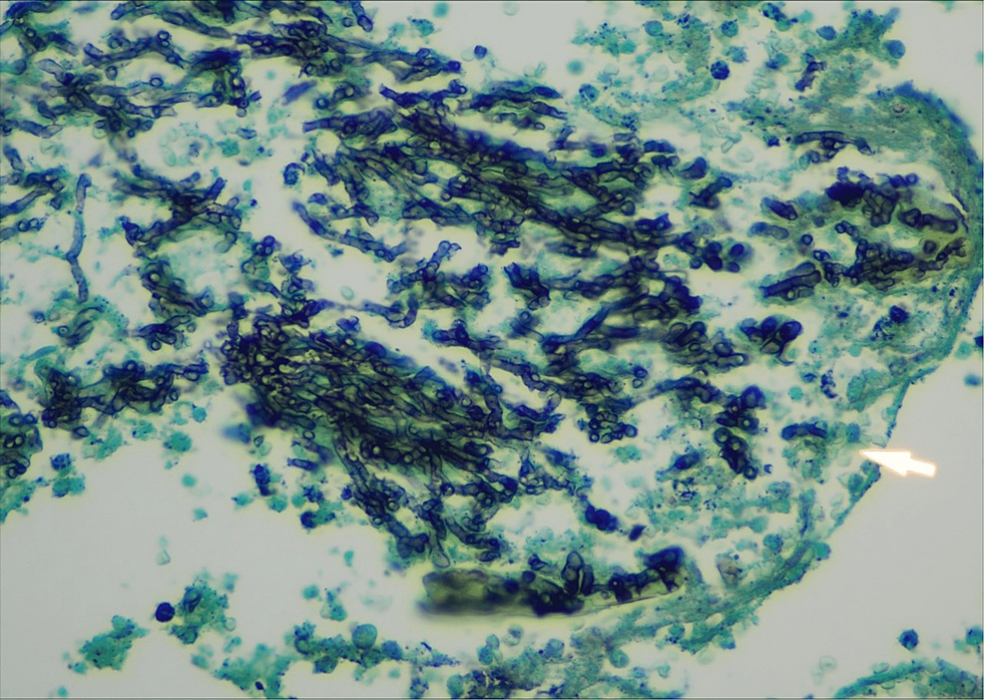
Gomori methenamine silver (GMS) stain of lung tissue showing fungal invasion consistent with IPA GMS stain of lung tissue showing fungal invasion consistent with IPA. The hyphae appear as thin, branching structures with a characteristic dichotomous branching pattern. The surrounding lung tissue shows signs of inflammation and necrosis.

Three months since his initial discharge, the patient returned to the hospital with a one-month history of worsening dyspnea, hemoptysis, and weight loss. CT chest revealed an intracavitary mass in the right lower lobe, suggestive of a fungus ball (Figure 4). Initial work-up, including acid-fast bacilli culture, Fungitell, galactomannan, blood cultures, MRSA by PCR, and mycobacterium smears, was all negative. Sputum culture revealed normal respiratory flora. The patient underwent a bronchoscopy due to worsening hemoptysis, and active bleeding was noted in the right lower lobe. The patient subsequently underwent right lower lobe wedge resection. Histopathological examination of the lung tissue revealed thin, branching hyphae and necrotizing granulomas without vascular invasion (Figure 5). Fungal culture of the lung tissue was positive for aspergillosis. A post-operative CT chest is shown below (Figure 6). 

The patient's clinical presentation, coupled with corroborative histopathological and microbiological evidence, solidified the diagnosis of IPA. Antifungal treatment was promptly initiated, incorporating the administration of voriconazole at a dosage of 200 mg, taken orally every 12 hours for a period of 21 days. Following this treatment regimen, the patient displayed notable clinical improvement, culminating in a discharge after a period of nine days. Upon transitioning to outpatient management, the patient continued to receive voriconazole therapy at the same dosage for an extended period of six months to manage chronic pulmonary aspergillosis, which was justified by ongoing symptoms such as mild hemoptysis, fatigue, and persistent cough. A follow-up chest CT scan was performed three months into the outpatient management period. The repeated imaging revealed complete resolution of the pulmonary cavitations, thus validating the sustained clinical improvement achieved through the antifungal therapy.

## Discussion

COVID-19 pneumonia, caused by the SARS-CoV-2 virus, is known to provoke persistent pulmonary symptoms and complications, encompassing chronic respiratory failure and a reduction in lung function [1-4]. An infrequent, yet notable complication is the emergence of cavitary lung disease after severe COVID-19 pneumonia [5,6], which can potentially escalate into fungal or bacterial superinfections.

The application of corticosteroids, despite their proven efficacy in minimizing mortality and the duration of mechanical ventilation in managing COVID-19 pneumonia, carries a risk of escalating opportunistic infections, especially among immunocompromised patients [7-9]. As found in a multinational study, COVID-19-associated pulmonary aspergillosis was seen to afflict up to 15% of ICU patients [10]. Furthermore, other studies demonstrated an association between IPA and the use of corticosteroids [11]. IPA, a severe and potentially fatal fungal infection instigated by Aspergillus species, is characterized by hallmark CT findings, such as nodules, halo signs, and air-crescent signs [12-14]. Additional CT findings may include consolidations, cavitations, pleural effusions, mediastinal lymphadenopathy, and vascular invasion.

Diagnosis of IPA ideally requires histopathological examination of lung tissue, typically obtained via thoracoscopic or open-lung biopsy [12]. The diagnosis is affirmed through the confluence of histopathological and microbiological findings, including the detection of fungal hyphae invasion and a positive culture for Aspergillus [12]. IPA treatment is primarily centered on antifungal therapy, with voriconazole considered the preferred first-line agent [12-15]. Depending on the specific circumstances, adjunctive measures, such as granulocyte colony-stimulating factor or surgical resection, may be utilized.

## Conclusions

This case demonstrates the uncommon sequelae of COVID-19 pneumonia, encompassing the development of cavitary lung disease and subsequent fungal superinfection following extended immunosuppressive therapy with corticosteroids. A careful approach should be adopted when considering prolonged corticosteroid therapy in patients demonstrating sequelae of COVID-19 pneumonia. Given the diagnostic challenges and necessity for a high index of suspicion, it is vital to closely monitor patients with COVID-19 pneumonia for the emergence of opportunistic infections. The timely identification of opportunistic infections, such as IPA evidenced in this case following COVID-19 pneumonia, carries profound implications for patient management. Especially in the context of extended corticosteroid therapy, it is imperative that healthcare professionals intervene with appropriate therapeutic measures, thereby mitigating adverse outcomes.
